# Roles of Tristetraprolin in Tumorigenesis

**DOI:** 10.3390/ijms19113384

**Published:** 2018-10-29

**Authors:** Jeong-Min Park, Tae-Hee Lee, Tae-Hong Kang

**Affiliations:** Department of Biological Science, Dong-A University, Busan 49315, Korea; zmpark@donga.ac.kr (J.-M.P.); thlee@donga.ac.kr (T.-H.L.)

**Keywords:** tristetraprolin (TTP), tumorigenesis, posttranscriptional regulation, adenosine and uridine-rich elements (AREs)

## Abstract

Genetic loss or mutations in tumor suppressor genes promote tumorigenesis. The prospective tumor suppressor tristetraprolin (TTP) has been shown to negatively regulate tumorigenesis through destabilizing the messenger RNAs of critical genes implicated in both tumor onset and tumor progression. Regulation of TTP has therefore emerged as an important issue in tumorigenesis. Similar to other tumor suppressors, TTP expression is frequently downregualted in various human cancers, and its low expression is correlated with poor prognosis. Additionally, disruption in the regulation of TTP by various mechanisms results in the inactivation of TTP protein or altered TTP expression. A recent study showing alleviation of Myc-driven lymphomagenesis by the forced expression of TTP has shed light on new therapeutic avenues for cancer prevention and treatment through the restoration of TTP expression. In this review, we summarize key oncogenes subjected to the TTP-mediated mRNA degradation, and discuss how dysregulation of TTP can contribute to tumorigenesis. In addition, the control mechanism underlying TTP expression at the posttranscriptional and posttranslational levels will be discussed.

## 1. Introduction

**Figure 1:**
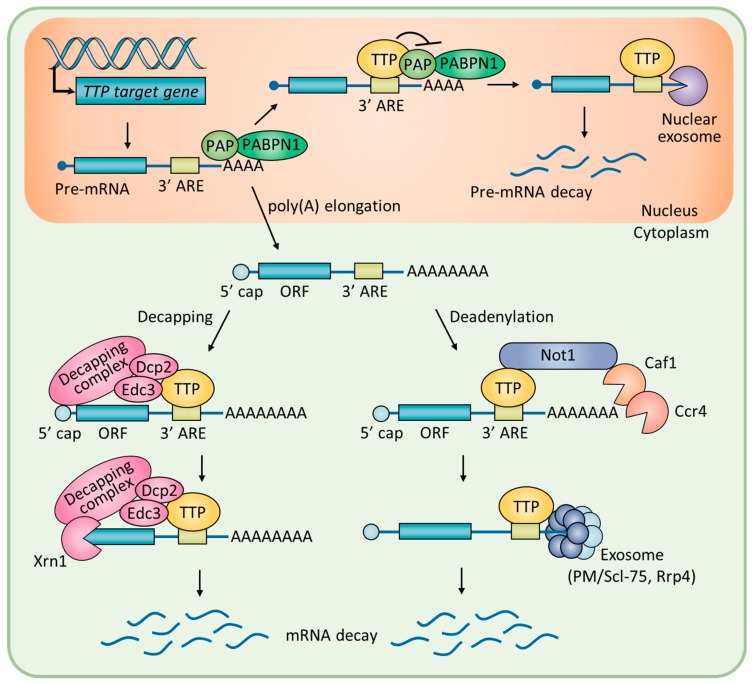
A schematic overview of posttranscriptional regulation of mRNA stability by TTP.

Posttranscriptional regulation of messenger RNA (mRNA) stability is essential for cells to rapidly respond to intracellular and extracellular stimuli [1,2]. The TPA-inducible sequence 11 (TIS11) family of RNA-binding proteins, composed of tristetraprolin (TTP) and butyrate response factors, modulates mRNA stability through direct binding to specific sequences located in the 3′ untranslated region (3′ UTR) of the target mRNA [3]. TTP, also known as TIS11A, G0/G1 switch regulatory protein 24 (GOS24), and growth factor-inducible nuclear protein NUP475, is encoded by the *ZFP36* gene. TTP contains a cysteine–cysteine-cysteine–histidine (CCCH) zinc finger motif for the recognition of cis-acting adenosine and uridine-rich elements (AREs) in the 3′ UTR of target mRNA [4,5]. As illustrated in Figure 1, binding of TTP to AREs generally facilitates the decay of the mRNA by means of recruiting enzymes for the rapid shortening of the poly(A) tail [6]. For instance, TTP interacts with the carbon catabolite repressor protein 4 (Ccr4)-negative on TATA (Not1) deadenylase complex, the exosome components polymyositis/systemic sclerosis 75 (PM/Scl-75), and ribosomal RNA processing 4 (Rrp4) to hydrolyze the poly(A) tail in a processive manner [7]. Alternatively, TTP interacts with poly(A)-binding protein nuclear 1 (PABPN1) in the nucleus to inhibit 3′-polyadenylation of pre-mRNA [8]. The 5′ to 3′ degradation of mRNA is processed by a decapping complex, which includes mRNA-decapping enzyme 2 (Dcp2), enhancer of mRNA-decapping protein 3 (Edc3), and 5′–3′ exoribonuclease (Xrn1), which interact with TTP [9,10].

The physiological importance of TTP in posttranscriptional coordination has been observed in TTP-deficient mice. These mice develop a complex syndrome of inflammatory arthritis, dermatitis, cachexia, autoimmunity, and myeloid hyperplasia [11]. These symptoms are recapitulated in the wild-type tumor necrosis factor alpha (TNF-α) transgenic and *TNF-α*^Δ*ARE*^ mice [12,13]. Indeed, TTP has been shown to accelerate the degradation of *TNF-α* mRNA via direct binding to the ARE in the 3′ UTR of *TNF-α* mRNA [14]. 

It was revealed that approximately 16% of human protein-coding genes have at least one consensus motif of an ARE in their 3′ UTR [15]. Many of these genes are implicated in immune responses and tumorigenesis [16,17]. Importantly, TTP has been shown to negatively regulate tumorigenesis by destabilizing its target mRNA linked to tumor onset and progression [18,19]. Thus, dysregulation of TTP has been regarded as an important issue in tumorigenesis [20]. In this review, we describe the current understanding of TTP’s roles in tumorigenesis, with a particular focus on the roles of TTP’s target genes during tumorigenesis. We summarize key oncogenes and tumor-associated genes subjected to TTP-mediated mRNA decay, and discuss how dysregulation of this process potentially contributes to tumorigenesis.

## 2. Oncogenes and Tumor Suppressor Genes Subjected to TTP-Mediated mRNA Decay

Tumorigenesis can be driven by the uncontrolled proliferation or the inappropriate survival of damaged cells due to the impairment of mRNA stability control of tumor-suppressor genes and oncogenes [21]. Table 1 shows the list of tumor-associated genes and their ARE sequences subjected to TTP-mediated mRNA degradation. The data indicate that TTP’s target mRNAs during tumorigenesis are predominantly oncogenes as opposed to tumor suppressors; 21 oncogenes were targets, as compared to three tumor suppressor genes, such as cyclin-dependent kinase inhibitor 1 (*CDKN1A*), large tumor suppressor kinase 2 (*LATS2*), and aryl-hydrocarbon receptor repressor (*AHRR*) [22,23,24].

**Figure 2:**
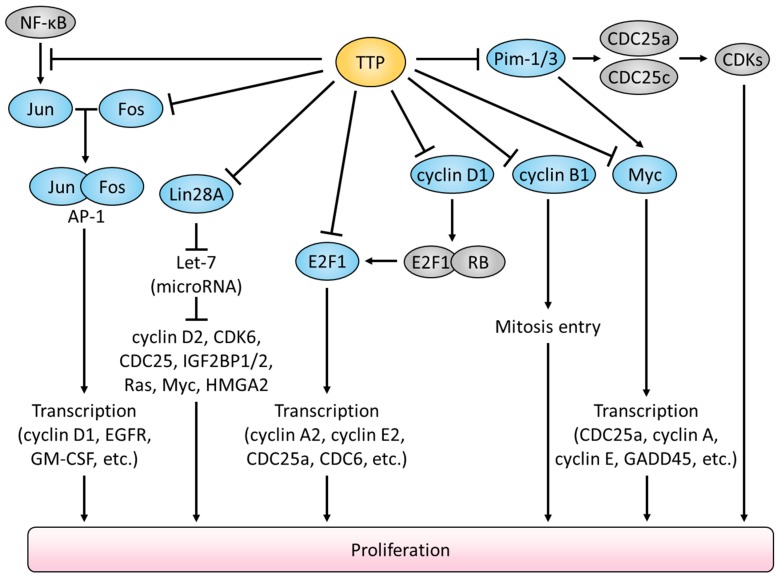
Attenuation of cellular proliferation by TTP-mediated suppression of oncogenic signalings.

TTP has been shown to prevent malignant proliferation by suppressing the expression of genes for cell-cycle progression and cellular proliferation depicted in Figure 2. Among these, *CCNB1* (cyclin B1) is the key oncogenic driver whose overexpression itself leads to the chronic proliferation of cancer cells [45]. Previous studies reported that high CCNB1 expression levels were detected in various cancers such as breast, colon, and non-small cell lung cancer [46,47,48]. Ectopic overexpression of TTP suppressed CCNB1 expression but depletion of TTP promoted the accumulation of *CCNB1* mRNA in human lung cancer cells [30]. This is because the ARE motif in *CCNB1* 3′ UTR is subjected to TTP-mediated degradation [25]. High expression of *CCND1* (cyclin D1) also correlates with tumor onset and tumor progression [49]. A recent study showed that treatment with the mechanistic target of rapamycin kinase (mTOR) inhibitor, rapamycin, induced rapid *CCND1* mRNA decay due to the increased TTP expression in glioblastoma cells [31]. CCND1 binding with cyclin dependent kinase 4 (CDK4) or CDK6 is necessary for the G1/S transition [50]. The active CDK4/6 phosphorylates retinoblastoma 1 (RB), which results in the release of E2F1 [51]. Subsequently, E2F1 initiates the expression of genes required for the cell cycle transition [52]. E2F1 also contains three AREs in its 3′ UTR [35]. In the meantime, the *PIM-1* oncogene is also subjected to TTP-mediated mRNA decay. PIM-1 facilitates cell cycle progression via activating *CDC25a* and *CDC25c* oncogenes [53]. In pancreatic cancer, low TTP expression was correlated with high PIM-1 expression, and patients with such gene expression profiles showed unfavorable survival rates [54]. 

In addition, by suppressing the expression of lin-28 homolog A (Lin28A), TTP can increase the expression of the tumor suppressor microRNA (miRNA) let-7, whose expression is negatively regulated by Lin28A [29,55]. The miRNA let-7 has been implicated in the regulation of gene transcription including high mobility group A2 (HMGA2) [56]. HMGA2 is frequently upregulated in multiple cancers, and is associated with both malignant and benign tumor formation [57]. The bioinformatic analysis discovered that 3′ UTR of *HMGA2* mRNA contains the hairpin structure termed HMGA2-sh, which is further processed to a HMGA2-sh-3p20 fragment through the action of Drosha and Dicer [37]. Interestingly, HMGA2-sh-3p20 elevated HMGA2 expression in hepatoma cells by means of preventing TTP binding to the *HMGA* mRNA [37]. Thus, HMGA2-sh-3p20 facilitates hepatocarcinogenesis by antagonizing the TTP-mediated decay of *HMGA2* mRNA. 

The collapse of the homeostatic balance between cell death and cell proliferation is a hallmark of cancer [58]. Simultaneous overexpression of the anti-apoptotic protein BCL2 and the *Myc* oncogene induces lymphoma [59]. By downregulating the expression of both genes, TTP has been shown to alleviate Myc-driven lymphomagenesis [27,60]. IAP (inhibitors of apoptosis proteins) family anti-apoptotic protein BIRC3 and XIAP are also under control by TTP-mediated mRNA decay [28,43]. Therefore, loss of TTP function would confer resistance to cancer cells against apoptotic stimulus, and promotes cancer cell viability due to the impairment of the destabilizing of anti-apoptotic gene expression. 

Aside from its canonical posttranscriptional role, TTP also has been implicated in the regulation of gene expression at the transcriptional level by participating in the nuclear factor kappa-light-chain-enhancer of activated B cells (NF-κB) pathway [39,61,62]. By blocking the nuclear import of NF-κB/p65, TTP suppresses the NF-κB-mediated transcription of oncogenes, including *c-Jun* [63]. c-Jun and c-Fos form the AP-1 early response transcription factor that promotes cell-cycle progression [64]. The stability of *c-Fos* mRNA is subjected to TTP-mediated posttranscriptional control [36]. Thereby, TTP regulates the activity of oncogenic AP-1 both at the transcriptional and posttranscriptional levels. 

## 3. Roles of TTP in Tumor Progression 

**Figure 3:**
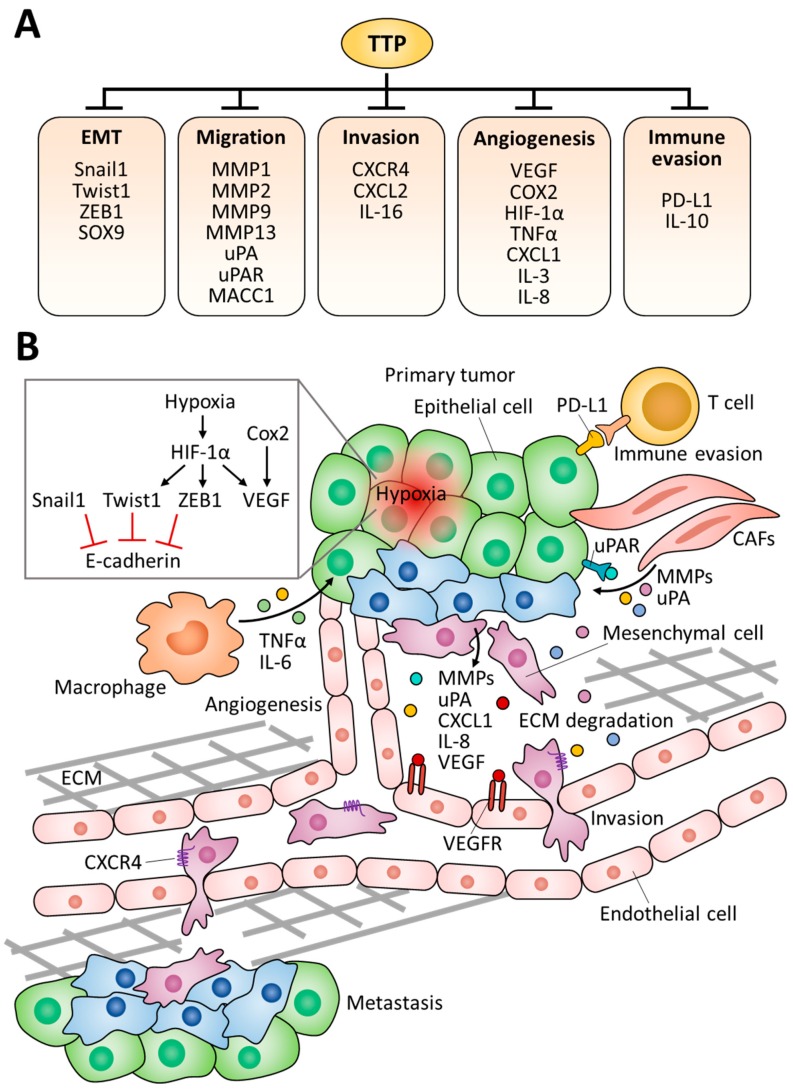
Suppressive roles of TTP in tumor progression. (**A**) TTP targets involved in the malignancy of tumors. EMT; epithelial-mesenchymal transition. (**B**) EMT and metastatic mechanisms driven by TTP target genes. CAFs; carcinoma-associated fibroblasts, ECM; extracellular matrix, VEGFR; vascular endothelial growth factor receptors.

Recent studies have revealed novel TTP targets involved in the malignancy of tumors, such as epithelial-mesenchymal transition (EMT), invasion, and metastasis (Figure 3). Based on the gene expression profiles from 80 patient samples (23 normal colon mucosa, 30 primary colon carcinoma, and 27 liver metastases), lower TTP expression was detected in primary tumors as compared to normal mucosa [41]. Furthermore, TTP expression was remarkably downregulated in metastatic tumors as compared to primary tumors, suggesting the possibility that TTP is engaged in the EMT process. Consistent with this notion, recent studies have reported that TTP facilitates the mRNA decay of EMT marker genes including *Snail1* (zinc finger protein snail 1), *Twist1* (twist-related protein 1), *ZEB1* (zinc finger E-box binding homeobox 1), *MMP-2* (matrix metalloproteinase 2), and MMP-9 [41,44,65]. 

The molecular signature of low E-cadherin, high vimentin, and high *N*-cadherin is an indicator of cells undergoing EMT; this feature was also detected in circulating tumor cells [66]. NIH:OVCAR3 (ovarian adenocarcinoma) and HT29 (colorectal adenocarcinoma) cells with high TTP levels exhibited high E-cadherin, low *N*-cadherin, and low vimentin, whereas low TTP-expressing SKOV3 (ovarian adenocarcinoma) and H1299 (non-small lung carcinoma) cells displayed low E-cadherin expression [44]. Snail1, Twist1, and ZEB1 are transcription factors for the transcriptional repression of E-cadherin [67]. TTP binding to the ARE within the 3′ UTR of these three genes triggered their mRNA decay [44]. In the meantime, loss of TTP increased the growth rate and migration capability of colorectal cancer cells due to the upregulation of ZEB1, sex-determining region Y box 9 (SOX9), and metastasis-associated in colon cancer 1 (MACC1) [41]. Furthermore, one of the most significant alterations underlying colorectal cancer is the constitutive activation of the T-cell factor (Tcf)/β-catenin signaling, and the administration of Tcf/β-catenin inhibitor FH535 derepressed TTP expression [41]. Collectively, TTP downregulates Snail1, Twist1, ZEB1, SOX9, and MACC1 expression at the posttranscriptional level to inhibit EMT. Thus, the recovery of TTP expression seems to be promising to suppress EMT in some types of human cancers.

EMT facilitates the reorganization of the extracellular matrix (ECM), since many EMT-inducing factors activate the expression of MMPs [68]. MMPs induce ECM degradation and allow tumor cells to migrate out of the primary tumor to form metastases [69]. Specifically, MMP-1 is an interstitial collagenase that decomposes collagen types I, II, and III, and MMP-13 breaks down type II collagen more efficiently than types I and III [70]. MMP-2, along with MMP-9, cleaves type-IV collagen, which is the most abundant component of the basement membrane of which breakdown is a critical step in the invasion and metastatic progression of cancer cells [70]. An invasion experiment recapitulating the oral mucosa showed that the suppression of TTP activity gives rise to an accelerated invasion rate of head and neck cancer cells due to the secretion of MMP-2, MMP-9, and interleukin-6 (IL-6) [65]. Mechanistically, p38-mediated phosphorylation and the inactivation of TTP upregulated the stability of *MMP-2*, *MMP-9*, and *IL-6* transcripts [65]. Another study showed that ectopic re-expression of TTP in breast cancer cells attenuated the invasion rate because TTP suppressed MMP-1 and MMP-13 expression [71]. Urokinase-type plasminogen activator (uPA) and its specific receptor urokinase plasminogen activator receptor (uPAR) are also implicated in the degradation of the ECM [72]. Overexpression of uPA and uPAR has been observed in invasive glioblastomas [73], and the ectopic expression of TTP alleviated the invasiveness of these cancer cells by suppressing the expression of both uPA and uPAR [74,75]. In addition, a recent report showed that the upregulated TTP expression led to significant downregulation of uPA and MMP-9 protein expression in breast cancer [76]. Taken together, uPA and uPAR are physiological targets of TTP in various cancer types, and the concept of TTP-mediated downregulation of uPA and uPAR seems to be promising to attenuate the malignancy of tumors [75].

Cancers develop in complex tissue environments known as tumor microenvironments, and these affect the growth and metastasis of tumor cells [77]. Tissues undergoing chronic inflammation due to the deregulation of the microenvironment generally exhibit a high incidence of cancer [78]. According to recent studies, programmed death-ligand 1 (PD-L1) is a novel target for TTP in gastric, lung, and colon cancer cells [79,80]. The expression of PD-L1 is essential for the development and functional maintenance of regulatory T cells [81], and its mRNA stability is negatively regulated by TTP [80]. Consequently, restoration of TTP expression enhanced anti-tumor immunity in a PD-L1-dependent manner [80]. Neoplastic cells are strongly influenced by the stroma, including surrounding and infiltrating cells. Immune cell infiltration into the tumor is an important determinant of tumor progression, and TTP depletion increases infiltration of monocytes/macrophages into the tumors [82]. IL-16 was identified as a critical TTP-regulated factor that contributes to the migration of immune cells [82]. IL-16 expression was increased in TTP-deficient 3D tumor spheroids, and elevated IL-16 levels enhanced the infiltration of monocytes into tumor spheroids [82]. Apparently, further studies are needed to determine the direct effect of TTP on IL-16 expression, but it seems clear that the loss of TTP allows immune cells within the microenvironment to promote tumor growth.

Tumor cells require a dedicated blood supply to obtain oxygen and nutrients for their maintenance and growth, and vascular endothelial growth factor (VEGF) is a crucial regulator of pathological angiogenesis [83]. TTP can bind to *VEGF* mRNA 3′ UTR and induce *VEGF* mRNA degradation [84]. Higher VEGF levels were detected in colorectal adenocarcinoma, as compared to normal tissues [85]. Cyclooxygenase 2 (COX-2) is also an important mediator of angiogenesis by facilitating the production of VEGF and BCL2 [86]. TTP binds between the nucleotides 3125 and 3232 in the 3′ UTR of *COX-2* mRNA and induces mRNA destabilization [87]. In colorectal cancer cells, low expression of TTP was responsible for the increased expression of COX-2 and VEGF, while overexpression of TTP in colon cancer cells markedly decreased the expression of both genes [88]. Moreover, cytokines related to tumor angiogenesis such as IL-3, IL-8, and TNF-α were reported as TTP targets which are suppressed by the way of ARE-mediated decay [32,89,90]. In contrast, TTP has been shown to increase human inducible nitric oxide synthase (iNOS) mRNA stability. TTP did not bind to human iNOS mRNA directly, but TTP destabilized the KH-type splicing regulatory protein (KSRP), which is responsible for iNOS mRNA decay, by facilitating recruitment of the exosome [91].

Although the precise mechanisms that determine the directional movement of tumor cells to distant sites are not well understood, this movement pattern seems similar to the chemokine-mediated leukocytes movement [92]. The expression of C-X-C motif chemokine receptor 4 (CXCR4) is low or absent in normal tissues, while it is highly expressed in various types of cancer, including colorectal cancer, ovarian cancer, and breast cancer [93,94], and the CXCR4 level was inversely correlated with TTP expression [34]. It has been revealed that CXCR4 is a TTP target containing a functional ARE in its 3′ UTR, and thus, induction of TTP results in the compromised CXCR4-mediated invasion and migration [34]. Furthermore, TTP depletion increased the production of several chemokines, such as C-X-C motif chemokine ligand 1 (CXCL1), CXCL2, and CXCL8 (also known as IL-8), which are involved in melanoma pathogenesis and angiogenesis [32,33,95]. Taken together, TTP has the ability to repress tumor metastasis by regulating chemokine-mediated migratory signaling. 

As the tumor grows, consumption of nutrients and oxygen around it lead to a state of nutrient and oxygen deprivation [96]. Subsequently, hypoxia induces hypoxia-inducible factor 1α (HIF-1α), an important transcription factor involved in angiogenesis, leading to angiogenesis that allows nutrients to the microenvironment around tumor tissue [97]. Importantly, TTP expression was induced in hypoxic cells, and the overexpression of TTP repressed the hypoxic induction of HIF-1α protein in colorectal cancer cells [98]. Thus, it was proposed that cancer cells may benefit from the downregulation of TTP, which subsequently increases HIF-1α expression and assists with the adaptation of cancer cells to hypoxia.

## 4. Regulation of TTP Expression in Normal and Cancer Cells

Recent research has demonstrated that TTP is abnormally expressed in various human malignancies [60,85,99,100,101,102,103,104,105]. TTP was initially identified as a member of immediate early response genes that are rapidly induced by the stimulation of insulin [106], serum [107], or mitogen [108,109] in quiescent fibroblasts. Serum-stimulated *TTP* mRNA induction was dependent on consensus binding sites for several transcription factors, such as early growth response protein 1 (EGR1), specificity protein 1 (SP-1), and activator protein 1 (AP-2) in the 5′-proximal region of the TTP promoter [110]. A few studies have shown that transcription of *TTP* was induced by growth-inhibitory cytokines during an inflammatory response. For instance, transforming growth factor beta 1 (TGF-β1)-induced TTP transcription was mediated by the binding of Smad3/4 transcription factors to the putative Smad-binding elements of the TTP promoter in human T cells [111]. Parallel to this, TTP expression was necessary for TGF-β1-dependent growth inhibition in normal intestinal epithelium [112]. In addition, the TTP promoter contains putative binding sites for signal transducer and activator of transcription (STAT) proteins. Indeed, STAT1, STAT3, and STAT6 were recruited to these sites, and induced *TTP* gene transcription under stimulation by different cytokines. Interferon gamma (IFN-γ)-induced STAT1 phosphorylation promoted *TTP* gene transcription [113]. IL-10-activated STAT3 or IL-4-activated STAT6 induced TTP expression through the janus kinase 1 (JAK1) pathway [114,115]. Interestingly, *TTP* mRNA is highly unstable, and the rapid turnover of *TTP* mRNA is due to an auto-regulatory negative feedback loop [116,117].

**Figure 4:**
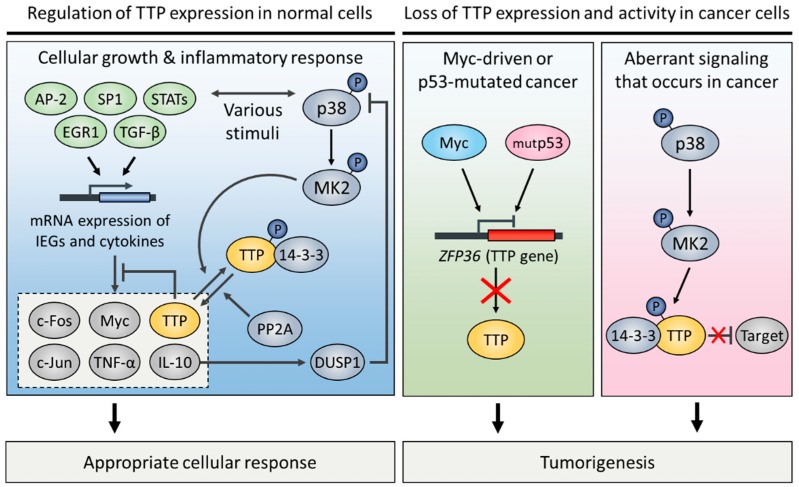
Regulation of TTP expression and activity in normal and cancer cells.

TTP expression is often deficient in several cancer types (Figure 4). Rounbehler and colleagues [60] found that TTP was expressed at low levels in Myc-expressing cancers including breast, colorectal, and metastatic prostate cancer. The 5′-proximal region of the *TTP* gene includes a putative initiator element (Inr) near the TATA box [110]. Myc directly inhibits the transcription of *TTP* through direct binding to the Inr. In contrast, the tumor suppressor p53 activates *TTP* mRNA expression in human cancer cells [55]. Wild-type p53 stimulated by the DNA-damaging agent doxorubicin was recruited to the TTP promoter to activate *TTP* transcription, whereas mutant p53 failed to induce *TTP* transcription [55]. The epigenetic gene silencing of the TTP promoter has been shown as an alternative way to regulate TTP expression [105,118,119]. For instance, TGF-β1-dependent Smad-binding region located in the TTP promoter has a specific single CpG site. In hepatocellular carcinoma cell lines, TTP expression was attenuated frequently by methylation of the CpG site [105]. MicroRNA-29a (miR-29a) targets the 3′ UTR of *TTP* mRNA, leading to the degradation of *TTP* mRNA in cancer cells [101,120]. In pancreatic and breast cancer, miR-29a-mediated *TTP* mRNA degradation was associated with EMT, and promoted tumor growth, invasion, and metastasis [101,120].

In addition to the loss of TTP expression, cells can become TTP deficient through a loss in TTP activity. Inactivation of TTP has been predominantly associated with its phosphorylation status. TTP can be phosphorylated by several kinases, including extracellular signal-regulated kinases (ERK) [121], p38 [121,122], c-Jun N-terminal kinases (JNK) [121], and v-akt murine thymoma viral oncogene (AKT) [31]. Among these, the p38 pathway is a major determinant for TTP activity but does not affect the protein level of TTP. p38 kinase phosphorylates and activates MAPK-activated protein kinase 2 (MK2); subsequently, MK2 phosphorylates TTP at serine 60 and 186 [123]. Subsequently, 14-3-3 proteins interact with phosphorylated TTP and inactivate it [31,124,125,126]. The TTP-14-3-3 complex cannot recruit the Ccr4-Not1 deadenylase complex, but has no impact on the binding affinity of ARE [124,125]. In addition, the interaction with 14-3-3 is required for cytoplasmic accumulation of TTP [126], which results in the inhibition of TTP’s role in the nucleus. Cytoplasmic TTP promotes the decay of mRNA containing AREs [18], whereas nuclear TTP functions as a transcriptional corepressor of NF-κB [39,127] and several nuclear receptors [128,129]. In breast cancer cells, ectopic overexpression of TTP was capable of repressing the transactivation activity of nuclear receptors, including estrogen receptor alpha (ERα), progesterone receptor (PR), glucocorticoid receptor (GR), and androgen receptor (AR), via physically interacting with these factors [128]. Mechanistically, nuclear TTP attenuates ERα transactivation by disrupting its interaction with steroid receptor coactivator 1 (SRC-1) [129]. 

The phosphorylation-induced TTP inactivation is reversed by two phosphatases. Protein phosphatase 2A (PP2A) directly dephosphorylates and reactivates TTP, but this results in a decrease in TTP protein stability [130,131,132,133,134]. Another phosphatase for TTP reactivation is dual specificity phosphatase 1 (DUSP1), that indirectly regulates TTP activity through the dephosphorylation of p38, which results in the inactivation of p38 kinase activity [135]. Several reports have indicated that a high level of the phosphorylated, inactive form of TTP was found in head and neck [65] and brain cancer cells [99,136]. Thus, the pharmacological application of a p38 inhibitor against these cancers may provide new ways to treat cancers containing hyperphosphorylated TTP. 

The first p38 inhibitors were identified in a screen for compounds that inhibited expression of TNF-α in human monocytic leukemia cell line [137]. In multiple myeloma and head and neck squamous cell carcinoma, p38 inhibitors were successfully used to limit tumor growth and angiogenesis, due indirectly to TTP-mediated inhibition of cytokine secretion [138,139]. p38 inhibitor also attenuates progression of malignant gliomas by inhibition of TTP phosphorylation [99]. Dufies and colleagues reported that p38 inhibitors may be a promising adjuvant therapy in cancer. Sunitinib, known as a first-line treatment for metastatic renal cell carcinoma, leads to patient relapse by p38 activation. While sunitinib mainly targets the host blood vessels via the inhibition of VEGF receptors, the mechanism of patient relapse is associated with increased lymphangiogenesis and lymph node metastasis via induction of *VEGFC* mRNA expression through p38-mediated inactivation of TTP. In renal cancer cells, the p38 inhibitor reduces the sunitinib-dependent increase in the *VEGFC* mRNA [140]. Several independent groups have identified effective drug candidates targeting TTP for anticancer therapies. For instance, Sorafenib targeting v-Raf murine sarcoma viral oncogene homolog B (B-Raf) kinase triggers re-expression of TTP in melanoma cells via the inhibition of B-Raf-dependent ERK activity [95]. Gambogic acid, the main active compound of *Gamboge hanburyi*, also induces upregulation of TTP expression through ERK inactivation, and efficiently inhibits the progression of colorectal cancer cells [141]. Histone deacetylase (HDAC) inhibitors in colorectal cancer cells induce TTP expression through activation of EGR1, which promote its binding affinity to ARE, and thus, reduce cell growth and angiogenesis [118,119]. Resveratrol, a natural polyphenolic compound present in many plant species, including grapes, peanuts, and berries, inhibits cell growth through TTP upregulation in several cancers, including breast [142], colorectal [29] and brain cancer [74]. Molecular activators of PP2A enhance the anti-inflammatory function of TTP in lung cancer cells, and thus, provide pharmacotherapeutic strategies to chronic inflammation-mediated cancer [133]. In addition, treatment with MK2 inhibitor triggers apoptosis in hepatocellular carcinoma. TTP knockdown rescued these cells from apoptosis in the presence of MK2 inhibitor, suggesting that the MK2-mediated TTP inactivation plays a role in cell survival of hepatocellular carcinoma [26]. These studies increase the understanding of the anti-cancer effects of various compounds and the molecular basis for further applications of therapeutic agents targeting TTP in clinical cancer therapy.

## 5. Conclusions 

The role of TTP as a key factor in posttranscriptional gene regulation has been established, especially with regard to its function in promoting mRNA decay of ARE-containing genes, including oncogenes and cancer-related cytokines. What has become more obvious is that TTP participates extensively in gene regulatory networks for tumor suppression. Cumulative evidence was provided that the loss of TTP expression or function was closely related with tumor onset and tumor progression, and presented poor outcomes of cancer patients. Based on current knowledge, many factors and signal pathways have been identified to regulate TTP at the transcriptional, posttranscriptional, and posttranslational level. The abnormal expression or activity of these factors consequently affected TTP’s expression or function. Therefore, endeavors for searching molecular pathways or chemical compounds upregulating TTP expression or activity will pave the way for potentially attractive therapeutics for cancer treatment.

## Figures and Tables

**Table 1:** List of oncogenes and tumor suppressor genes subjected to the ARE-mediated mRNA decay by TTP.

Gene Symbol	ARE Sequences	Regulation by TTP			
		ARE Binding ^1^	3′ UTR Binding ^2^	mRNA Decay ^3^	Ref.
*AHRR*	TTCTGGCCTCTGGGCATTTATGGATTTAAGACCAGGATGGTATTTCAGAAGCTT	O	O	O	[23]
*AKT-1*	TTTTTTTACAACATTCAACTTTAGT	O	ND	O	[25,26]
*BCL2*	ATTTATTTATTTA	ND	O	O	[27]
*BIRC3* (*cIAP2*)	TTTGGTTTCCTTAAAATTTTTATTTATTTACAACTCAAAAAACATTGTTTTG	O	O	O	[28,29]
*CCNB1* (*cyclin B1*)	TTATTTACTTTTACCACTATTTAAG	O	O	O	[25,30]
*CCND1* (*cyclin D1*)	TTATTATATTCCGTAGGTAGATGTG, ACATAATATATTCTATTTTTATACTCT	O	O	O	[25,31]
*CDKN1A* (*p21*)	TAGTCTCAGTTTGTGTGTCTTAATTATTATTTGTGTTTTAATTTAAACACCTCCT	O	O	O	[24]
*CXCL1*	TCTTCTATTTATTTATTTATTCATTAGTT	O	O	O	[25,32]
*CXCL2* (*MIP-2*)	CACACTCTCCCATTATATTTATTG	O	ND	O	[25,33]
*CXCR4*	ACTTATTTATATAAATTTTTTTTG	O	O	O	[25,34]
*E2F1*	CTTTAATGGAGCGTTATTTATTTATCGAGGCCTCTTTG	O	O	O	[29,35]
*FOS* (*c-Fos*)	TAATTTATTTATT	ND	O	O	[36]
*HMGA2*	TGTAATTTAATGA	ND	O	O	[37]
*IFN-γ*	CTATTTATTAATATTTAA	O	O	O	[38]
*JUN* (*c-Jun*)	TTCTCTATTAGACTTTAGAAA, AGCACTCTGAGTTTACCATTTG	O	ND	ND	[25,39]
*LATS2*	TTCAAATTAGTATGATTCCTATTTAAAGTGATTTATATTTGAGTAAAAAGTTCAA	O	O	O	[22]
*Lin28A*	TTTTATTTATTTG	O	O	O	[29,40]
*MACC1*	TATAATTTAATAT	ND	O	O	[41]
*MYC* (*Myc*)	AATTTCAATCCTAGTATATAGTACCTAGTATTATAGGTACTATAAACCCTAATTTTTTTTATTTAA	O	O	O	[25,31]
*PIM-1*	CCTGGAGGTCAATGTTATGTATTTATTTATTTATTTATTTGGTTCCCTTCCTATTCC	O	O	O	[42]
*PIM-3*	TTTAATTTATTTG	ND	O	O	[43]
*SNAI1* (*Snail1*)	GTTATATGTACAGTTTATTGATATTCAATAAAGCAGTTAATTTATATATTAAAAA	O	O	O	[44]
*XIAP*	CAAATTTATTTTATTTATTTAATT	O	O	O	[25,43]

^1^ O: experimentally determined ARE sequences; ND: not determined experimentally, the predicted ARE sequences are from ARED-Plus web source; ^2,3^ O: experimentally confirmed; ND: not determined experimentally.

## Abbreviations

3′ UTR	3′ untranslated regions
AHRR	Aryl-hydrocarbon receptor repressor
AKT-1	AKT serine/threonine kinase 1
AP-1	Activator protein 1
AR	Androgen receptor
AREs	Adenosine and uridine-rich elements
BCL2	B-cell CLL/Lymphoma 2
BIRC3	Baculoviral IAP repeat containing 3
B-Raf	v-Raf murine sarcoma viral oncogene homolog B
CAFs	Carcinoma-associated fibroblasts
CCCH	Cysteine–cysteine-cysteine–histidine
CCNB1	Cyclin B1
CCND1	Cyclin D1
Ccr4	Carbon catabolite repressor protein 4
CDC25A	Cell division cycle 25 homolog A
CDK4	Cyclin dependent kinase 4
CDK6	Cyclin dependent kinase 6
CDKN1A	Cyclin dependent kinase inhibitor 1A
COX-2	Cyclooxygenase 2
CXCL1	C-X-C motif chemokine ligand 1
CXCL2	C-X-C motif chemokine ligand 2
CXCR4	C-X-C motif chemokine receptor 4
Dcp2	mRNA-decapping enzyme 2
DUSP1	Dual specificity phosphatase 1
E2F1	E2F transcription factor 1
ECM	Extracellular matrix
EGR1	Early growth response protein 1
Edc3	Enhancer of mRNA-decapping protein 3
EMT	Epithelial-mesenchymal transition
ERK	Extracellular signal-regulated kinases
ERα	Estrogen receptor alpha
FOS	FBJ murine osteosarcoma viral oncogene homolog
GOS24	G0/G1 switch regulatory protein 24
GR	Glucocorticoid receptor
HDAC	Histone deacetylase
HIF-1α	Hypoxia-inducible factor 1α
HMGA2	High mobility group A2
IAP	Inhibitors of apoptosis proteins
IFN-γ	Interferon gamma
IL-6	Interleukin-6
iNOS	inducible nitric oxide synthase
Inr	Initiator element
JAK1	Janus kinase 1
JNK	c-Jun N-terminal kinases
JUN	v-jun avian sarcoma virus 17 oncogene homolog
KSRP	KH-type splicing regulatory protein
LATS2	Large tumor suppressor kinase 2
Lin28A	Lin-28 homolog A
MACC1	Metastasis associated in colon cancer 1
MAPK	Mitogen-activated protein kinase
miR-29a	MicroRNA-29a
MK2	MAPK-activated protein kinase 2
MMP-13	Matrix metalloproteinase 13
MMP-2	Matrix metalloproteinase 2
MMP-9	Matrix metalloproteinase 9
mRNA	Messenger RNAs
Myc	v-myc avian myelocytomatosis viral oncogene homolog
NF-κB	Nuclear factor kappa-light-chain-enhancer of activated B cells
Not1	Negative on TATA 1
NUP475	Growth factor-inducible nuclear protein NUP475
PABPN1	Poly-A-binding protein nuclear 1
PD-L1	Programmed death-ligand 1
PIM-1	Proto-oncogene serine/threonine-protein kinase pim 1
PM/Scl-75	Polymyositis/systemic sclerosis 75
PP2A	Protein phosphatase 2A
PR	Progesterone receptor
RB	Retinoblastoma 1
Rrp4	Ribosomal RNA processing 4
SNAI1	Zinc finger protein snail 1
SOX9	Sex-determining region Y box 9
SP-1	Specificity protein 1
SRC-1	Steroid receptor coactivator 1
STAT	Signal transducer and activator of transcription
Tcf	T-cell factor
TGF-β1	Transforming growth factor beta 1
TIS	12-*O*-tetradecanoylphorbol-13-acetate (TPA)-induced sequence
TNF-α	Tumor necrosis factor alpha
TTP	Tristetraprolin
Twist1	Twist-related protein 1
uPA	Urokinase-type plasminogen activator
uPAR	Urokinase plasminogen activator receptor
VEGF	Vascular endothelial growth factor
VEGFR	Vascular endothelial growth factor receptors
XIAP	X-linked inhibitor of apoptosis
Xrn1	5′-3′ exoribonuclease
ZEB1	Zinc finger E-box binding homeobox 1
ZFP36	Zinc finger protein 36

## References

[1] Guhaniyogi J., Brewer G. (2001). Regulation of mRNA stability in mammalian cells. Gene.

[2] Park J.M., Kang T.H. (2016). Transcriptional and posttranslational regulation of nucleotide excision repair: The guardian of the genome against ultraviolet radiation. Int. J. Mol. Sci..

[3] Sanduja S., Blanco F.F., Dixon D.A. (2011). The roles of TTP and BRF proteins in regulated mRNA decay. Wiley Interdiscip. Rev. RNA.

[4] Fu M.G., Blackshear P.J. (2017). RNA-binding proteins in immune regulation: A focus on ccch zinc finger proteins. Nat. Rev. Immunol..

[5] Hudson B.P., Martinez-Yamout M.A., Dyson H.J., Wright P.E. (2004). Recognition of the mRNA AU-rich element by the zinc finger domain of tis11d. Nat. Struct. Mol. Biol..

[6] Lai W.S., Carballo E., Thorn J.M., Kennington E.A., Blackshear P.J. (2000). Interactions of CCCH zinc finger proteins with mRNA—Binding of tristetraprolin-related zinc finger proteins to au-rich elements and destabilization of mRNA. J. Biol. Chem..

[7] Sandler H., Kreth J., Timmers H.T.M., Stoecklin G. (2011). Not1 mediates recruitment of the deadenylase CAF1 to mRNAs targeted for degradation by tristetraprolin. Nucleic Acids Res..

[8] Su Y.L., Wang S.C., Chiang P.Y., Lin N.Y., Shen Y.F., Chang G.D., Chang C.J. (2012). Tristetraprolin inhibits poly(a)-tail synthesis in nuclear mRNA that contains AU-rich elements by interacting with poly(a)-binding protein nuclear 1. PLoS ONE.

[9] Eulalio A., Behm-Ansmant I., Izaurralde E. (2007). P bodies: At the crossroads of post-transcriptional pathways. Nat. Rev. Mol. Cell Biol..

[10] Fenger-Gron M., Fillman C., Norrild B., Lykke-Andersen J. (2005). Multiple processing body factors and the are binding protein ttp activate mRNA decapping. Mol. Cell.

[11] Taylor G.A., Carballo E., Lee D.M., Lai W.S., Thompson M.J., Patel D.D., Schenkman D.I., Gilkeson G.S., Broxmeyer H.E., Haynes B.F. (1996). A pathogenetic role for tnf alpha in the syndrome of cachexia, arthritis, and autoimmunity resulting from tristetraprolin (TTP) deficiency. Immunity.

[12] Probert L., Akassoglou K., Alexopoulou L., Douni E., Haralambous S., Hill S., Kassiotis G., Kontoyiannis D., Pasparakis M., Plows D. (1996). Dissection of the pathologies induced by transmembrane and wild-type tumor necrosis factor in transgenic mice. J. Leukoc. Biol..

[13] Kontoyiannis D., Pasparakis M., Pizarro T.T., Cominelli F., Kollias G. (1999). Impaired on/off regulation of tnf biosynthesis in mice lacking tnf AU-rich elements: Implications for joint and gut-associated immunopathologies. Immunity.

[14] Lai W.S., Carballo E., Strum J.R., Kennington E.A., Phillips R.S., Blackshear P.J. (1999). Evidence that tristetraprolin binds to au-rich elements and promotes the deadenylation and destabilization of tumor necrosis factor alpha mRNA. Mol. Cell. Biol..

[15] Gruber A.R., Fallmann J., Kratochvill F., Kovarik P., Hofacker I.L. (2011). Aresite: A database for the comprehensive investigation of au-rich elements. Nucleic Acids Res..

[16] Khabar K.S.A. (2017). Hallmarks of cancer and AU-rich elements. Wiley Interdiscip. Rev. RNA.

[17] Bisogno L.S., Keene J.D. (2018). RNA regulons in cancer and inflammation. Curr. Opin. Genet. Dev..

[18] Brooks S.A., Blackshear P.J. (2013). Tristetraprolin (TTP): Interactions with mRNA and proteins, and current thoughts on mechanisms of action. Biochim. Biophys. Acta Gene Regul. Mech..

[19] Guo J., Qu H.H., Chen Y., Xia J.Z. (2017). The role of RNA-binding protein tristetraprolin in cancer and immunity. Med. Oncol..

[20] Wang H., Ding N.N., Guo J., Xia J.Z., Ruan Y.L. (2016). Dysregulation of TTP and hur plays an important role in cancers. Tumor Biol..

[21] Esquela-Kerscher A., Slack F.J. (2006). Oncomirs—Micrornas with a role in cancer. Nat. Rev. Cancer.

[22] Lee H.H., Vo M.T., Kim H.J., Lee U.H., Kim C.W., Kim H.K., Ko M.S., Lee W.H., Cha S.J., Min Y.J. (2010). Stability of the lats2 tumor suppressor gene is regulated by tristetraprolin. J. Biol. Chem..

[23] Lee H.H., Kim W.T., Kim D.H., Park J.W., Kang T.H., Chung J.W., Leem S.H. (2013). Tristetraprolin suppresses ahrr expression through mRNA destabilization. FEBS Lett..

[24] Al-Haj L., Blackshear P.J., Khabar K.S.A. (2012). Regulation of P21/CIP1/WAF-1 mediated cell-cycle arrest by rnase l and tristetraprolin, and involvement of au-rich elements. Nucleic Acids Res..

[25] Mukherjee N., Jacobs N.C., Hafner M., Kennington E.A., Nusbaum J.D., Tuschl T., Blackshear P.J., Ohler U. (2014). Global target mRNA specification and regulation by the RNA-binding protein ZFP36. Genome Biol..

[26] Tran D.D.H., Koch A., Allister A., Saran S., Ewald F., Koch M., Nashan B., Tamura T. (2016). Treatment with mapkap2 (MK2) inhibitor and DNA methylation inhibitor, 5-aza dc, synergistically triggers apoptosis in hepatocellular carcinoma (HCC) via tristetraprolin (TTP). Cell. Signal..

[27] Park S.B., Lee J.H., Jeong W.W., Kim Y.H., Cha H.J., Joe Y., Chung H.T., Cho W.J., Do J.W., Lee B.J. (2015). TTP mediates cisplatin-induced apoptosis of head and neck cancer cells by down-regulating the expression of Bcl-2. J. Chemother..

[28] Kim C.W., Kim H.K., Vo M.T., Lee H.H., Kim H.J., Min Y.J., Cho W.J., Park J.W. (2010). Tristetraprolin controls the stability of CIAP2 mRNA through binding to the 3′ UTR of CIAP2 mRNA. Biochem. Biophys. Res. Commun..

[29] Lee S.R., Jin H., Kim W.T., Kim W.J., Kim S.Z., Leem S.H., Kim S.M. (2018). Tristetraprolin activation by resveratrol inhibits the proliferation and metastasis of colorectal cancer cells. Int. J. Oncol..

[30] Zheng X.T., Xiao X.Q. (2015). Sodium butyrate down-regulates tristetraprolin-mediated cyclin b1 expression independent of the formation of processing bodies. Int. J. Biochem. Cell Biol..

[31] Marderosian M., Sharma A., Funk A.P., Vartanian R., Masri J., Jo O.D., Gera J.F. (2006). Tristetraprolin regulates Cyclin D1 and c-Myc mRNA stability in response to rapamycin in an AKT-dependent manner via p38 MAPK signaling. Oncogene.

[32] Datta S., Biswas R., Novotny M., Pavicic P.G., Herjan T., Mandal P., Hamilton T.A. (2008). Tristetraprolin regulates cxcl1 (KC) mRNA stability. J. Immunol..

[33] Jalonen U., Nieminen R., Vuolteenaho K., Kankaanranta H., Moilanen E. (2006). Down-regulation of tristetraprolin expression results in enhanced IL-12 and MIP-2 production and reduced MIP-3alpha synthesis in activated macrophages. Mediat. Inflamm..

[34] Al-Souhibani N., Al-Ghamdi M., Al-Ahmadi W., Khabar K.S.A. (2014). Posttranscriptional control of the chemokine receptor CXCR4 expression in cancer cells. Carcinogenesis.

[35] Lee H.H., Lee S.R., Leem S.H. (2014). Tristetraprolin regulates prostate cancer cell growth through suppression of E2F1. J. Microbiol. Biotechnol..

[36] Amit I., Citri A., Shay T., Lu Y.L., Katz M., Zhang F., Tarcic G., Siwak D., Lahad J., Jacob-Hirsch J. (2007). A module of negative feedback regulators defines growth factor signaling. Nat. Genet..

[37] Wang Y., Chen F.Q., Yang Z., Zhao M., Zhang S.Q., Gao Y., Feng J.Y., Yang G., Zhang W.Y., Ye L.H. (2017). The fragment HMGA2-sh-3p20 from HMGA2 mRNA 3′UTR promotes the growth of hepatoma cells by upregulating HMGA2. Sci. Rep..

[38] Ogilvie R.L., SternJohn J.R., Rattenbacher B., Vlasova I.A., Williams D.A., Hau H.H., Blackshear P.J., Bohjanen P.R. (2009). Tristetraprolin mediates interferon-gamma mRNA decay. J. Biol. Chem..

[39] Xu L., Ning H., Gu L., Wang Q., Lu W., Peng H., Cui W., Ying B., Ross C.R., Wilson G.M. (2015). Tristetraprolin induces cell cycle arrest in breast tumor cells through targeting AP-1/c-jun and nf-kappab pathway. Oncotarget.

[40] Kim C.W., Vo M.T., Kim H.K., Lee H.H., Yoon N.A., Lee B.J., Min Y.J., Joo W.D., Cha H.J., Park J.W. (2012). Ectopic over-expression of tristetraprolin in human cancer cells promotes biogenesis of let-7 by down-regulation of Lin28. Nucleic Acids Res..

[41] Montorsi L., Guizzetti F., Alecci C., Caporali A., Martello A., Atene C.G., Parenti S., Pizzini S., Zanovello P., Bortoluzzi S. (2016). Loss of zfp36 expression in colorectal cancer correlates to wnt/beta-catenin activity and enhances epithelial-to-mesenchymal transition through upregulation of zeb1, sox9 and macc1. Oncotarget.

[42] Kim H.K., Kim C.W., Vo M.T., Lee H.H., Lee J.Y., Yoon N.A., Lee C.Y., Moon C.H., Min Y.J., Park J.W. (2012). Expression of proviral integration site for moloney murine leukemia virus 1 (pim-1) is post-transcriptionally regulated by tristetraprolin in cancer cells. J. Biol. Chem..

[43] Selmi T., Martello A., Vignudelli T., Ferrari E., Grande A., Gemelli C., Salomoni P., Ferrari S., Zanocco-Marani T. (2012). zfp36 Expression impairs glioblastoma cell lines viability and invasiveness by targeting multiple signal transduction pathways. Cell Cycle.

[44] Yoon N.A., Jo H.G., Lee U.H., Park J.H., Yoon J.E., Ryu J., Kang S.S., Min Y.J., Ju S.A., Seo E.H. (2016). Tristetraprolin suppresses the EMT through the down-regulation of twist1 and snail1 in cancer cells. Oncotarget.

[45] Yuan J., Kramer A., Matthess Y., Yan R., Spankuch B., Gatje R., Knecht R., Kaufmann M., Strebhardt K. (2006). Stable gene silencing of Cyclin b1 in tumor cells increases susceptibility to taxol and leads to growth arrest in vivo. Oncogene.

[46] Kawamoto H., Koizumi H., Uchikoshi T. (1997). Expression of the G2-M checkpoint regulators Cyclin b1 and cdc2 in nonmalignant and malignant human breast lesions: Immunocytochemical and quantitative image analyses. Am. J. Pathol..

[47] Wang A., Yoshimi N., Ino N., Tanaka T., Mori H. (1997). Overexpression of Cyclin b1 in human colorectal cancers. J. Cancer Res. Clin. Oncol..

[48] Soria J.C., Jang S.J., Khuri F.R., Hassan K., Liu D., Hong W.K., Mao L. (2000). Overexpression of Cyclin b1 in early-stage non-small cell lung cancer and its clinical implication. Cancer Res..

[49] Diehl J.A. (2002). Cycling to cancer with Cyclin d1. Cancer Biol. Ther..

[50] Musgrove E.A., Caldon C.E., Barraclough J., Stone A., Sutherland R.L. (2011). Cyclin d as a therapeutic target in cancer. Nat. Rev. Cancer.

[51] Wang S., Nath N., Fusaro G., Chellappan S. (1999). Rb and prohibitin target distinct regions of e2f1 for repression and respond to different upstream signals. Mol. Cell. Biol..

[52] Lee S.R., Roh Y.G., Kim S.K., Lee J.S., Seol S.Y., Lee H.H., Kim W.T., Kim W.J., Heo J., Cha H.J. (2015). Activation of ezh2 and suz12 regulated by E2F1 predicts the disease progression and aggressive characteristics of bladder cancer. Clin. Cancer Res..

[53] Zhang Y., Wang Z., Magnuson N.S. (2007). Pim-1 kinase-dependent phosphorylation of p21Cip1/WAF1 regulates its stability and cellular localization in H1299 cells. Mol. Cancer Res..

[54] Wei Z.R., Liang C., Feng D., Cheng Y.J., Wang W.M., Yang D.J., Wang Y.X., Cai Q.P. (2016). Low tristetraprolin expression promotes cell proliferation and predicts poor patients outcome in pancreatic cancer. Oncotarget.

[55] Lee J.Y., Kim H.J., Yoon N.A., Lee W.H., Min Y.J., Ko B.K., Lee B.J., Lee A., Cha H.J., Cho W.J. (2013). Tumor suppressor p53 plays a key role in induction of both tristetraprolin and let-7 in human cancer cells. Nucleic Acids Res..

[56] Lujambio A., Lowe S.W. (2012). The microcosmos of cancer. Nature.

[57] Wu J.J., Zhang S.Z., Shan J.L., Hu Z.J., Liu X.Y., Chen L.R., Ren X.C., Yao L.F., Sheng H.Q., Li L. (2016). Elevated HMGA2 expression is associated with cancer aggressiveness and predicts poor outcome in breast cancer. Cancer Lett..

[58] Cooper J.P., Youle R.J. (2012). Balancing cell growth and death. Curr. Opin. Cell Biol..

[59] Otake Y., Soundararajan S., Sengupta T.K., Kio E.A., Smith J.C., Pineda-Roman M., Stuart R.K., Spicer E.K., Fernandes D.J. (2007). Overexpression of nucleolin in chronic lymphocytic leukemia cells induces stabilization of bcl2 mRNA. Blood.

[60] Rounbehler R.J., Fallahi M., Yang C., Steeves M.A., Li W., Doherty J.R., Schaub F.X., Sanduja S., Dixon D.A., Blackshear P.J. (2012). Tristetraprolin impairs Myc-induced lymphoma and abolishes the malignant state. Cell.

[61] Gu L., Ning H., Qian X., Huang Q., Hou R., Almourani R., Fu M., Blackshear P.J., Liu J. (2013). Suppression of il-12 production by tristetraprolin through blocking nf-kcyb nuclear translocation. J. Immunol..

[62] Chen Y.L., Jiang Y.W., Su Y.L., Lee S.C., Chang M.S., Chang C.J. (2013). Transcriptional regulation of tristetraprolin by NF-kappaB signaling in lps-stimulated macrophages. Mol. Biol. Rep..

[63] Schichl Y.M., Resch U., Hofer-Warbinek R., de Martin R. (2009). Tristetraprolin impairs NF-kappaB/p65 nuclear translocation. J. Biol. Chem..

[64] Schreiber M., Kolbus A., Piu F., Szabowski A., Mohle-Steinlein U., Tian J., Karin M., Angel P., Wagner E.F. (1999). Control of cell cycle progression by c-jun is p53 dependent. Genes Dev..

[65] Van Tubergen E.A., Banerjee R., Liu M., Broek R.V., Light E., Kuo S., Feinberg S.E., Willis A.L., Wolf G., Carey T. (2013). Inactivation or loss of TTP promotes invasion in head and neck cancer via transcript stabilization and secretion of MMP9, MMP2, and IL-6. Clin. Cancer Res..

[66] Roussos E.T., Keckesova Z., Haley J.D., Epstein D.M., Weinberg R.A., Condeelis J.S. (2010). AACR special conference on epithelial-mesenchymal transition and cancer progression and treatment. Cancer Res..

[67] Nieto M.A., Huang R.Y.J., Jackson R.A., Thiery J.P. (2016). EMT: 2016. Cell.

[68] Radisky E.S., Radisky D.C. (2010). Matrix metalloproteinase-induced epithelial-mesenchymal transition in breast cancer. J. Mammary Gland Biol. Neoplasia.

[69] Egeblad M., Werb Z. (2002). New functions for the matrix metalloproteinases in cancer progression. Nat. Rev. Cancer.

[70] Kessenbrock K., Plaks V., Werb Z. (2010). Matrix metalloproteinases: Regulators of the tumor microenvironment. Cell.

[71] Al-Ahmadi W., Al-Ghamdi M., Al-Souhibani N., Khabar K.S.A. (2013). miR-29a inhibition normalizes hur over-expression and aberrant AU-rich mRNA stability in invasive cancer. J. Pathol..

[72] Rao J.S. (2003). Molecular mechanisms of glioma invasiveness: The role of proteases. Nat. Rev. Cancer.

[73] Yamamoto M., Ueno Y., Hayashi S., Fukushima T. (2002). The role of proteolysis in tumor invasiveness in glioblastoma and metastatic brain tumors. Anticancer Res..

[74] Ryu J., Yoon N.A., Seong H., Jeong J.Y., Kang S., Park N., Choi J., Lee D.H., Roh G.S., Kim H.J. (2015). Resveratrol induces glioma cell apoptosis through activation of tristetraprolin. Mol. Cells.

[75] Ryu J., Yoon N.A., Lee Y.K., Jeong J.Y., Kang S., Seong H., Choi J., Park N., Kim N., Cho W.J. (2015). Tristetraprolin inhibits the growth of human glioma cells through downregulation of urokinase plasminogen activator/urokinase plasminogen activator receptor mRNAs. Mol. Cells.

[76] Xiong T., Liu X.W., Huang X.L., Xu X.F., Xie W.Q., Zhang S.J., Tu J. (2018). Tristetraprolin: A novel target of diallyl disulfide that inhibits the progression of breast cancer. Oncol. Lett..

[77] Quail D.F., Joyce J.A. (2013). Microenvironmental regulation of tumor progression and metastasis. Nat. Med..

[78] Grivennikov S.I., Greten F.R., Karin M. (2010). Immunity, inflammation, and cancer. Cell.

[79] Guo J., Qu H.H., Shan T., Chen Y.G., Chen Y., Xia J.Z. (2018). Tristetraprolin overexpression in gastric cancer cells suppresses PD-L1 expression and inhibits tumor progression by enhancing antitumor immunity. Mol. Cells.

[80] Coelho M.A., Trecesson S.D., Rana S., Zecchin D., Moore C., Molina-Arcas M., East P., Spencer-Dene B., Nye E., Barnouin K. (2017). Oncogenic Ras signaling promotes tumor immunoresistance by stabilizing PD-L1 mRNA. Immunity.

[81] Francisco L.M., Salinas V.H., Brown K.E., Vanguri V.K., Freeman G.J., Kuchroo V.K., Sharpe A.H. (2009). PD-L1 regulates the development, maintenance, and function of induced regulatory T cells. J. Exp. Med..

[82] Milke L., Schulz K., Weigert A., Sha W.X., Schmid T., Brune B. (2013). Depletion of tristetraprolin in breast cancer cells increases interleukin-16 expression and promotes tumor infiltration with monocytes/macrophages. Carcinogenesis.

[83] Neufeld G., Cohen T., Gengrinovitch S., Poltorak Z. (1999). Vascular endothelial growth factor (VEGF) and its receptors. FASEB J..

[84] Essafi-Benkhadir K., Onesto C., Stebe E., Moroni C., Pages G. (2007). Tristetraprolin inhibits ras-dependent tumor vascularization by inducing vascular endothelial growth factor mRNA degradation. Mol. Biol. Cell.

[85] Lee H.H., Son Y.J., Lee W.H., Park Y.W., Chae S.W., Cho W.J., Kim Y.M., Choi H.J., Choi D.H., Jung S.W. (2010). Tristetraprolin regulates expression of VEGF and tumorigenesis in human colon cancer. Int. J. Cancer.

[86] Gately S., Li W.W. (2004). Multiple roles of cox-2 in tumor angiogenesis: A target for antiangiogenic therapy. Semin. Oncol..

[87] Sawaoka H., Dixon D.A., Oates J.A., Boutaud O. (2003). Tristetraprolin binds to the 3′-untranslated region of cyclooxygenase-2 mRNA. A polyadenylation variant in a cancer cell line lacks the binding site. J. Biol. Chem..

[88] Cha H.J., Lee H.H., Chae S.W., Cho W.J., Kim Y.M., Choi H.J., Choi D.H., Jung S.W., Min Y.J., Lee B.J. (2011). Tristetraprolin downregulates the expression of both VEGF and COX-2 in human colon cancer. Hepatogastroenterology.

[89] Hau H.H., Walsh R.J., Ogilvie R.L., Williams D.A., Reilly C.S., Bohjanen P.R. (2007). Tristetraprolin recruits functional mRNA decay complexes to are sequences. J. Cell. Biochem..

[90] Winzen R., Thakur B.K., Dittrich-Breiholz O., Shah M., Redich N., Dhamija S., Kracht M., Holtmann H. (2007). Functional analysis of ksrp interaction with the au-rich element of interleukin-8 and identification of inflammatory mRNA targets. Mol. Cell. Biol..

[91] Fechir M., Linker K., Pautz A., Hubrich T., Forstermann U., Rodriguez-Pascual F., Kleinert H. (2005). Tristetraprolin regulates the expression of the human inducible nitric-oxide synthase gene. Mol. Pharmacol..

[92] Zhu Q.C., Han X.D., Peng J.Y., Qin H.L., Wang Y. (2012). The role of CXC chemokines and their receptors in the progression and treatment of tumors. J. Mol. Histol..

[93] Balkwill F. (2004). Cancer and the chemokine network. Nat. Rev. Cancer.

[94] Zeelenberg I.S., Ruuls-Van Stalle L., Roos E. (2003). The chemokine receptor CXCR4 is required for outgrowth of colon carcinoma micrometastases. Cancer Res..

[95] Bourcier C., Griseri P., Grepin R., Bertolotto C., Mazure N., Pages G. (2011). Constitutive erk activity induces downregulation of tristetraprolin, a major protein controlling interleukin8/CXCL8 mRNA stability in melanoma cells. Am. J. Physiol. Cell Physiol..

[96] Harris A.L. (2002). Hypoxia—A key regulatory factor in tumour growth. Nat. Rev. Cancer.

[97] Giatromanolaki A., Harris A.L. (2001). Tumour hypoxia, hypoxia signaling pathways and hypoxia inducible factor expression in human cancer. Anticancer Res..

[98] Kim T.W., Yim S., Choi B.J., Jang Y., Lee J.J., Sohn B.H., Yoo H.S., Il Yeom Y., Park K.C. (2010). Tristetraprolin regulates the stability of hif-1 alpha mRNA during prolonged hypoxia. Biochem. Biophys. Res. Commun..

[99] Suswam E., Li Y., Zhang X., Gillespie G.Y., Li X., Shacka J.J., Lu L., Zheng L., King P.H. (2008). Tristetraprolin down-regulates interleukin-8 and vascular endothelial growth factor in malignant glioma cells. Cancer Res.

[100] Brennan S.E., Kuwano Y., Alkharouf N., Blackshear P.J., Gorospe M., Wilson G.M. (2009). The mRNA-destabilizing protein tristetraprolin is suppressed in many cancers, altering tumorigenic phenotypes and patient prognosis. Cancer Res..

[101] Gebeshuber C.A., Zatloukal K., Martinez J. (2009). Mir-29a suppresses tristetraprolin, which is a regulator of epithelial polarity and metastasis. EMBO Rep..

[102] Carrick D.M., Blackshear P.J. (2007). Comparative expression of tristetraprolin (TTP) family member transcripts in normal human tissues and cancer cell lines. Arch. Biochem. Biophys..

[103] Sanduja S., Kaza V., Dixon D.A. (2009). The mRNA decay factor tristetraprolin (ttp) induces senescence in human papillomavirus-transformed cervical cancer cells by targeting E6-AP ubiquitin ligase. Aging.

[104] Young L.E., Sanduja S., Bemis-Standoli K., Pena E.A., Price R.L., Dixon D.A. (2009). The mRNA binding proteins hur and tristetraprolin regulate cyclooxygenase 2 expression during colon carcinogenesis. Gastroenterology.

[105] Sohn B.H., Park I.Y., Lee J.J., Yang S.J., Jang Y.J., Park K.C., Kim D.J., Lee D.C., Sohn H.A., Kim T.W. (2010). Functional switching of TGF-beta1 signaling in liver cancer via epigenetic modulation of a single CPG site in ttp promoter. Gastroenterology.

[106] Lai W.S., Stumpo D.J., Blackshear P.J. (1990). Rapid insulin-stimulated accumulation of an mRNA encoding a proline-rich protein. J. Biol. Chem..

[107] DuBois R.N., McLane M.W., Ryder K., Lau L.F., Nathans D. (1990). A growth factor-inducible nuclear protein with a novel cysteine/histidine repetitive sequence. J. Biol. Chem..

[108] Lim R.W., Varnum B.C., Herschman H.R. (1987). Cloning of tetradecanoyl phorbol ester-induced ‘primary response’ sequences and their expression in density-arrested swiss 3T3 cells and a TPA non-proliferative variant. Oncogene.

[109] Gomperts M., Pascall J.C., Brown K.D. (1990). The nucleotide sequence of a cdna encoding an EGF-inducible gene indicates the existence of a new family of mitogen-induced genes. Oncogene.

[110] Lai W.S., Thompson M.J., Taylor G.A., Liu Y., Blackshear P.J. (1995). Promoter analysis of zfp-36, the mitogen-inducible gene encoding the zinc finger protein tristetraprolin. J. Biol. Chem..

[111] Ogawa K., Chen F., Kim Y.J., Chen Y. (2003). Transcriptional regulation of tristetraprolin by transforming growth factor-beta in human T cells. J. Biol. Chem..

[112] Blanco F.F., Sanduja S., Deane N.G., Blackshear P.J., Dixon D.A. (2014). Transforming growth factor beta regulates p-body formation through induction of the mRNA decay factor tristetraprolin. Mol. Cell. Biol..

[113] Sauer I., Schaljo B., Vogl C., Gattermeier I., Kolbe T., Muller M., Blackshear P.J., Kovarik P. (2006). Interferons limit inflammatory responses by induction of tristetraprolin. Blood.

[114] Gaba A., Grivennikov S.I., Do M.V., Stumpo D.J., Blackshear P.J., Karin M. (2012). Cutting edge: IL-10-mediated tristetraprolin induction is part of a feedback loop that controls macrophage STAT3 activation and cytokine production. J. Immunol..

[115] Suzuki K., Nakajima H., Ikeda K., Maezawa Y., Suto A., Takatori H., Saito Y., Iwamoto I. (2003). IL-4-STAT6 signaling induces tristetraprolin expression and inhibits TNF-alpha production in mast cells. J. Exp. Med..

[116] Tchen C.R., Brook M., Saklatvala J., Clark A.R. (2004). The stability of tristetraprolin mRNA is regulated by mitogen-activated protein kinase p38 and by tristetraprolin itself. J. Biol. Chem..

[117] Brooks S.A., Connolly J.E., Rigby W.F. (2004). The role of mRNA turnover in the regulation of tristetraprolin expression: Evidence for an extracellular signal-regulated kinase-specific, AU-rich element-dependent, autoregulatory pathway. J. Immunol..

[118] Sharma A., Bhat A.A., Krishnan M., Singh A.B., Dhawan P. (2013). Trichostatin-a modulates claudin-1 mRNA stability through the modulation of hu antigen r and tristetraprolin in colon cancer cells. Carcinogenesis.

[119] Sobolewski C., Sanduja S., Blanco F.F., Hu L., Dixon D.A. (2015). Histone deacetylase inhibitors activate tristetraprolin expression through induction of early growth response protein 1 (EGR1) in colorectal cancer cells. Biomolecules.

[120] Sun X.J., Liu B.Y., Yan S., Jiang T.H., Cheng H.Q., Jiang H.S., Cao Y., Mao A.W. (2015). MicroRNA-29a promotes pancreatic cancer growth by inhibiting tristetraprolin. Cell. Physiol. Biochem..

[121] Cao H., Dzineku F., Blackshear P.J. (2003). Expression and purification of recombinant tristetraprolin that can bind to tumor necrosis factor-alpha mRNA and serve as a substrate for mitogen-activated protein kinases. Arch. Biochem. Biophys..

[122] Carballo E., Cao H., Lai W.S., Kennington E.A., Campbell D., Blackshear P.J. (2001). Decreased sensitivity of tristetraprolin-deficient cells to p38 inhibitors suggests the involvement of tristetraprolin in the p38 signaling pathway. J. Biol. Chem..

[123] Rigby W.F., Roy K., Collins J., Rigby S., Connolly J.E., Bloch D.B., Brooks S.A. (2005). Structure/function analysis of tristetraprolin (TTP): P38 stress-activated protein kinase and lipopolysaccharide stimulation do not alter ttp function. J. Immunol..

[124] Marchese F.P., Aubareda A., Tudor C., Saklatvala J., Clark A.R., Dean J.L. (2010). Mapkap kinase 2 blocks tristetraprolin-directed mRNA decay by inhibiting CAF1 deadenylase recruitment. J. Biol. Chem..

[125] Clement S.L., Scheckel C., Stoecklin G., Lykke-Andersen J. (2011). Phosphorylation of tristetraprolin by MK2 impairs au-rich element mRNA decay by preventing deadenylase recruitment. Mol. Cell. Biol..

[126] Johnson B.A., Stehn J.R., Yaffe M.B., Blackwell T.K. (2002). Cytoplasmic localization of tristetraprolin involves 14-3-3-dependent and -independent mechanisms. J. Biol. Chem..

[127] Liang J., Lei T., Song Y., Yanes N., Qi Y., Fu M. (2009). RNA-destabilizing factor tristetraprolin negatively regulates NF-kappaB signaling. J. Biol. Chem..

[128] Barrios-Garcia T., Gomez-Romero V., Tecalco-Cruz A., Valadez-Graham V., Leon-Del-Rio A. (2016). Nuclear tristetraprolin acts as a corepressor of multiple steroid nuclear receptors in breast cancer cells. Mol. Genet. Metab. Rep..

[129] Barrios-Garcia T., Tecalco-Cruz A., Gomez-Romero V., Reyes-Carmona S., Meneses-Morales I., Leon-Del-Rio A. (2014). Tristetraprolin represses estrogen receptor alpha transactivation in breast cancer cells. J. Biol. Chem..

[130] Hitti E., Iakovleva T., Brook M., Deppenmeier S., Gruber A.D., Radzioch D., Clark A.R., Blackshear P.J., Kotlyarov A., Gaestel M. (2006). Mitogen-activated protein kinase-activated protein kinase 2 regulates tumor necrosis factor mRNA stability and translation mainly by altering tristetraprolin expression, stability, and binding to adenine/uridine-rich element. Mol. Cell. Biol..

[131] Cristobal I., Torrejon B., Madoz-Gurpide J., Rojo F., Garcia-Foncillas J. (2017). PP2A plays a key role in inflammation and cancer through tristetraprolin activation. Ann. Rheum. Dis..

[132] O’Neil J.D., Ammit A.J., Clark A.R. (2018). Mapk p38 regulates inflammatory gene expression via tristetraprolin: Doing good by stealth. Int. J. Biochem. Cell. Biol..

[133] Rahman M.M., Rumzhum N.N., Hansbro P.M., Morris J.C., Clark A.R., Verrills N.M., Ammit A.J. (2016). Activating protein phosphatase 2a (PP2A) enhances tristetraprolin (TTP) anti-inflammatory function in a549 lung epithelial cells. Cell Signal..

[134] Sun L., Stoecklin G., Van Way S., Hinkovska-Galcheva V., Guo R.F., Anderson P., Shanley T.P. (2007). Tristetraprolin (TTP)-14-3-3 complex formation protects ttp from dephosphorylation by protein phosphatase 2a and stabilizes tumor necrosis factor-alpha mRNA. J. Biol. Chem..

[135] Clark A.R., Dean J.L. (2016). The control of inflammation via the phosphorylation and dephosphorylation of tristetraprolin: A tale of two phosphatases. Biochem. Soc. Trans..

[136] Suswam E.A., Shacka J.J., Walker K., Lu L., Li X., Si Y., Zhang X., Zheng L., Nabors L.B., Cao H. (2013). Mutant tristetraprolin: A potent inhibitor of malignant glioma cell growth. J. Neurooncol..

[137] Lee J.C., Laydon J.T., McDonnell P.C., Gallagher T.F., Kumar S., Green D., McNulty D., Blumenthal M.J., Heys J.R., Landvatter S.W. (1994). A protein kinase involved in the regulation of inflammatory cytokine biosynthesis. Nature.

[138] Medicherla S., Reddy M., Ying J., Navas T.A., Li L., Nguyen A.N., Kerr I., Hanjarappa N., Protter A.A., Higgins L.S. (2008). P38alpha-selective map kinase inhibitor reduces tumor growth in mouse xenograft models of multiple myeloma. Anticancer Res..

[139] Banerjee R., Van Tubergen E.A., Scanlon C.S., Vander Broek R., Lints J.P., Liu M., Russo N., Inglehart R.C., Wang Y., Polverini P.J. (2014). The G protein-coupled receptor GALR2 promotes angiogenesis in head and neck cancer. Mol. Cancer Ther..

[140] Dufies M., Giuliano S., Ambrosetti D., Claren A., Ndiaye P.D., Mastri M., Moghrabi W., Cooley L.S., Ettaiche M., Chamorey E. (2017). Sunitinib stimulates expression of vegfc by tumor cells and promotes lymphangiogenesis in clear cell renal cell carcinomas. Cancer Res..

[141] Wei F., Zhang T., Yang Z., Wei J.C., Shen H.F., Xiao D., Wang Q., Yang P., Chen H.C., Hu H. (2018). Gambogic acid efficiently kills stem-like colorectal cancer cells by upregulating zfp36 expression. Cell. Physiol. Biochem..

[142] Li C., Tang C., He G. (2016). Tristetraprolin: A novel mediator of the anticancer properties of resveratrol. Genet. Mol. Res..

